# Commentary: Gut Antibody Deficiency in a Mouse Model of CVID Results in Spontaneous Development of a Gluten-Sensitive Enteropathy

**DOI:** 10.3389/fimmu.2020.01921

**Published:** 2020-08-27

**Authors:** Silje F. Jørgensen, Børre Fevang, Pål Aukrust

**Affiliations:** ^1^Division of Surgery, Inflammatory Diseases and Transplantation, Research Institute of Internal Medicine, Oslo University Hospital, Oslo, Norway; ^2^Section of Clinical Immunology and Infectious Diseases, Department of Rheumatology, Dermatology and Infectious Diseases, Oslo University Hospital, Oslo, Norway; ^3^Institute of Clinical Medicine, University of Oslo, Oslo, Norway

**Keywords:** CVID, mouse model, gut microbiota, enteropathy, celiac disease, primary immunodeficiency, dysbiosis, gluten sensitivity

The development and maturation of the immune system are dependent on the presence of the gut microbiota. This is illustrated by the fact that germ-free mice do not develop isolated lymphoid follicles and they are also deficient in secretory IgA. However, if these mice are colonized with commensal bacteria, both maturation of isolated lymphoid follicles and intestinal IgA secretion occur (1–3). Studies of immune deficient mice lacking both B and T cells (Rag^−/−^) have shown that these mice have considerably less diverse gut microbiota compared with Rag^+/−^ littermates, or with wild type animals raised in the same facility. Likewise, mice lacking B cells (Ighm^−/−^) or T cells (Cd3e^−/−^) also have reduced bacterial diversity and different phylogenic structure (4). Furthermore, murine models deficient of IgA (or reduced IgA production) also affect gut microbiota composition, resulting in an immense activation of the systemic immune system. These pioneering studies have shown that the absence of immune cells affect gut microbial composition and *vice versa*, and this bidirectional interaction is also linked to systemic inflammation (4–7).

It was therefore with great interest that we read the paper by Mohammed et al. exploring gut microbiota, gut permeability, and enteropathy in CD19^−/−^ mice (8). They found that gut microbial diversity was altered with an overgrowth of anaerobic bacteria in the gut of these B- cell deficient mice. This overgrowth corresponded with the development of an inflammatory enteropathy in the ileum, and increased gut mucosal permeability in CD19^−/−^ mice compared to wild type mice. Interestingly, the enteropathy found in these mice was reversed by a gluten-free diet, suggesting that the gastrointestinal enteropathy in CD19^−/−^ mice might be linked to enhanced gluten sensitivity.

Common Variable Immunodeficiency (CVID) is a disease characterized by both immunodeficiency, autoimmunity and systemic inflammation. It is estimated that 10–20% of CVID patients have a monogenic cause and that the remaining have a polygenic cause influenced by environmental and epigenetic factors (9). Shulzhenko et al. have previously explored gene expression profiles in intestinal biopsies from three patients with CVID, and have shown microbiota-induced changes that may affect B-cell development (10). We found similar changes in stool samples from 44 CVID patients with a large shift in the gut microbiota composition compared to controls. The diversity of the gut microbiota, i.e., number of bacterial species present, correlated negatively with the gut leakage marker, lipopolysaccharides, known to promote inflammatory responses in macrophages through Toll like receptors (11). Furthermore, Shulzhenko et al. showed upregulation of genes expressing immune function and inflammation, at the expense of genes expressing metabolic functions in gut biopsies from both B cell deficient mice and CVID patients (10). These findings suggest that in the presence of a functional immune system, the intestinal epithelium can concentrate on its metabolic functions. However, if the immune system is dysfunctional, the intestinal epithelium takes on some of the missing immune functions at the expense of its metabolic activity. We recognize that little data on CVID in experimental models exist, in part due to scarcity of adequate CVID mouse models. However, we have some concerns with the use of CD19^−/−^ mice as a disease model for CVID. In our opinion, the immunological and clinical phenotype of CVID are not adequately covered in a CD19^−/−^ mice model. CVID patients have, with a few exceptions, normal number of B cells and a more complex immunological phenotype that also affects T cells, monocytes/macrophages, and dendritic cells (12). The authors‘ rationale for using CD19^−/−^ mice as disease model for CVID is also argued by the fact that some CVID patients have mutation in *CD19* and thereby CD19 deficiency. This is a very rare cause of the monogenic cause of CVID, and was not found in any CVID patients in a recently published cohort of 469 patients (13). Instead, a wide range of monogenic causes were found in 10% of CVID patients including monogenic causes such as *LRBA* and *CTLA4* mutations, which are characterized by an immense T cell immune activation and impaired function of regulatory T cells. Therefore, this mouse model does not reflect monogenic causes of CVID, which are more complex than *CD19* mutations, and definitively not a polygenic cause of CVID. Of note, the above-mentioned study also had an extended cohort that consisted of 1,318 patients with Primary immunodeficiency. In this cohort *CD19* mutation was found in one patient with Primary antibody deficiency, not classified as CVID (13).

The apparent coexistence of CVID and celiac disease has previously been described (14), but it is not clear if the similar findings in biopsies from CVID patients and celiac disease patients have a common pathogenic mechanism. There is also a discrepancy in the literature regarding gluten avoidance in CVID patients with “celiac-like” disease (14–16). We have previously demonstrated that CVID patients with “celiac-like disease” and true celiac disease are different disease entities as assessed by gene-expression analysis from duodenal biopsies (17). Network analyses revealed that genes encoding molecules involved in Lipid metabolism, Small Molecule Biochemistry, Vitamin and Mineral Metabolism had the highest score when differentiating between these two disease entities. The finding by Mohammed et al., of reversal of enteropathy in CD19^−/−^ mice on gluten-free diet, is interesting, but since these mice do not reflect the immunological- or clinical phenotype of CVID, the results from this study cannot be transferred to the human disease, CVID. Particularly, the title of the paper could lead to misunderstandings, as CD19^−/−^ mice are not mentioned, but referred to as a CVID mouse model. We appreciate that the authors have discussed some of the problems with referring to CD19^−/−^ mice as a CVID mouse model in the Discussion, but the overall impression in the article is that CD19^−/−^ mice resemble CVID in humans.

That aside, we find the experiments in this paper to be of high quality. The findings linking B cell deficient mice to gut microbial changes that could be altered trough antibiotics and diet, and thereby effect gut leakage and enteropathy, are very interesting. It would been of great interest if the authors in further studies could explore any potential transcriptome changes before and after gluten avoidance that could point toward particular genes, molecules or networks involved in the apparent reversal of symptoms in these mice. In addition, the link between gut microbiota, immunodeficiency, and defects in lipid metabolism, is a great contribution to the literature and should be investigated further. Although CD19^−/−^ mice are suitable for studying the effect on B cell deficiency on gut mucosa, gut microbiota, and gut leakage, this model should, in our opinion, not be referred to as a CVID mouse model.

## Author Contributions

SJ, BF, and PA wrote the paper. All authors contributed to the article and approved the submitted version.

## Conflict of Interest

The authors declare that the research was conducted in the absence of any commercial or financial relationships that could be construed as a potential conflict of interest.
